# Assessing extracellular vesicles from bovine mammary gland epithelial cells cultured in FBS-free medium

**DOI:** 10.20517/evcna.2021.18

**Published:** 2021-12-31

**Authors:** Giulia Silvestrelli, Susanne E. Ulbrich, Mara D. Saenz-de-Juano

**Affiliations:** ETH Zurich, Animal Physiology, Animal Physiology, Department of Environmental System Science (D-USYS), Institute of Agricultural Sciences, Zurich 8092, Switzerland.

**Keywords:** Extracellular vesicles, bovine mammary gland epithelial cells, FBS-free medium

## Abstract

**Aim:**

Mammary gland extracellular vesicles (EVs) are found in both human and livestock milk. Our knowledge of the role of EVs in the mammary gland development, breast cancer and mastitis derives mainly from *in vitro* cell culture models. However, a commonly shared limitation is the use of fetal bovine serum (FBS) as a supplement, which naturally contains EVs. For this reason, the purpose of the study was to evaluate novel tools to investigate mammary gland EVs *in vitro* and in a FBS-free system.

**Methods:**

Primary bovine mammary epithelial cells (pbMECs) and a mammary gland alveolar epithelial cell line (MAC-T) were cultured in a chemically defined EV-free medium. To find a reliable EV isolation protocol from a starting cell conditioned medium (10 mL), we compared eight different methodologies by combining ultracentrifugation (UC), chemical precipitation (CP), size exclusion chromatography (SEC), and ultrafiltration (UF).

**Results:**

The medium formula sustained both pbMECs and MAC-T cell growth. Transmission electron microscopy revealed that we obtained EV-like particles in five out of eight protocols. The cleanest samples with the highest number of particles and detectable amounts of RNA were obtained by using UF-SEC-UC.

**Conclusion:**

Our chemically defined, FBS-free medium sustains the growth of both pbMECs and MAC-T and allows the isolation of EVs that are free from any contamination by UF-SEC-UC. In conclusion, we propose a new culture system and EVs isolation protocols for further research on mammary epithelial EVs.

## INTRODUCTION

Mammary gland extracellular vesicles (EVs) participate in many physiological processes of the mammary gland such as development^[1,2]^ and regulation of epithelial cells polarity^[3]^. They are likewise involved in pathological conditions, including mastitis^[4,5]^ and breast cancer *in primis*, where EVs are implicated in its onset^[6]^, metastasis^[7]^, and drug resistance^[8,9]^. Mammary gland EVs are also found in the milk of human^[10]^ and many livestock species such as cattle^[11,12]^, buffalo^[13]^, and goat^[14]^.

Our knowledge of the molecular mechanisms underlying EVs biogenesis, release, uptake, and their effect on recipient cells, derives mainly from *in vitro* cell culture models. Many studies on human cell lines have investigated the role of EVs in mammary gland communication in the context of cancer^[9,15,16]^ or the maintenance of epithelial cells polarity^[3]^. In mice, EVs play an important role in mammary gland development^[3]^ and involution, and EVs promote the recognition and clearance of apoptotic bodies from macrophages^[2]^.

While most of the published studies focus on humans and mice, only a few studies investigated EVs directly in bovine mammary gland culture models, from primary cells^[17] ^or cell lines^[18]^. Bovine milk EVs are heterogenous and comprise many EVs subtypes including exosomes (40-100 nm) and microvesicles (100-1000 nm). In addition, milk EVs are biologically active; they are transferred to the newborn and are taken up from intestinal cells^[11]^. To date, their role is still not known in cell-cell communication within the alveolus of the mammary gland. Besides, the origin of milk EVs is not fully clear, despite recent publications suggesting that they are produced by the milk-secreting epithelial cells, the lactocytes^[19,20]^. Of note, autocrine and paracrine communication within the bovine mammary gland alveolus is important in all its developmental steps^[21]^, and, in this frame, EVs might also participate in this intense communication.

Cell culture systems allow more options and flexibility to investigate EVs mediated communication as compared to *in vivo* systems. However, they usually share a limitation, namely the use of fetal bovine serum (FBS) as a supplement. FBS naturally contains EVs that can interfere with the experimental setting and outcome^[22]^; therefore, many studies use commercial or home-made EV-depleted FBS, also in bovine mammary epithelial cells’ EVs research^[23]^. However, the removal of EVs from FBS is never complete^[22]^ and alters the FBS effect on cells, affecting cell growth^[24]^, differentiation^[25]^, and response to pathogens^[26,27]^. A period of 24-48 h of starvation from FBS affects the culture conditions as well, leading to cell cycle arrest^[28]^ and reducing the background production of cytokines^[29]^, which can cause misinterpretation of cell phenotype and experimental outcome. For these reasons, it is recommended to culture cells directly in FBS-free systems and chemically defined media. Besides the type of medium, it is critical to collect enough EVs for downstream analyses. For this reason, the volume of the starting material usually ranges between 20 and 250 mL^[30-32]^. Another factor affecting the yield and purity of the EVs sample is the isolation procedure^[33]^.

Few cell lines from the bovine mammary gland exist, with BME-UV1^[34]^ and MAC-T^[35]^ as the most commonly known to date. The use of pure epithelial cell lines prevents contamination from fibroblasts often occurring in primary cultures, which can also be avoided by preplating^[36]^, short trypsinization and isolating epithelial cells from milk^[37]^. MAC-T cells are considered a proper model for mammary gland development and lactation^[38]^, as they maintain the capacity to express milk proteins^[39]^ and are responsive to hormonal stimulation^[34]^. However, cell lines tend to lose the phenotypes of the original tissue^[40]^ and they are set to grow in specific culture conditions, therefore the change of culture conditions can also alter their characteristics.

To gain novel insights on the EVs local communication of the bovine mammary gland, *in vitro* models compiling the guidelines for EVs research are in need. For this reason, in the current study we: (1) developed a chemically defined FBS-free culture that supports the growth of both primary MEC and MAC-T cell line; and (2) compared eight different EVs isolation protocols from only 10 mL of conditioned medium regarding the quantity and size distribution of EVs for further downstream analyses.

## METHODS

### Primary cells isolation and culture

Mammary glands from lactating cows were collected at a local slaughterhouse postmortem and transported on crushed ice to the lab. Only mammary glands that did not display fibrosis, abnormal cell growth, and signs of mastitis (e.g., redness or hardness) were used to set the cultures. Tissue pieces of ~10 g were pooled from two to four cows each and washed in 70% ethanol, and then cold PBS (Gibco, Thermo Fisher, USA) with antibiotics. Tissues were further minced in ~2 mm^3^ pieces and washed six times in cold PBS with antibiotics. They were digested in 0.5 mg/mL collagenase IV (Sigma-Aldrich, USA), 0.5 mg/mL dispase II (Sigma), 5 μg/mL insulin (Sigma), antibiotics, and antimycotics in HBSS (Gibco) buffer for 2 h at 37 °C while gently shaking. The suspension was filtered through a metal mesh to remove larger tissue pieces and then centrifuged at 500 x*g* for 5 min. The pellet was washed twice in PBS and cells were seeded on Nunclon Delta surface dishes (Thermo Fisher) and kept at 37.5 °C 7% CO_2_. For the preplating, freshly isolated cells were plated and left for 1 h in the incubator. Then, the medium and cells that were not attached yet were transferred into a new plate. The medium was changed every 2-3 days until 80% confluence; cells were then subpassaged every 3-4 days. Cells at P1 were analyzed at 80% confluency. Cells were kept in DMEM/F12 (Gibco), containing 50 μg/mL gentamicin (Sigma) and 2.5 μg/mL amphotericin B (Sigma), and supplemented with: (1) FBS 10%; or (2) 1:50 B27 (Gibco), 5 μg/mL insulin (Sigma), 5 μg/mL hydrocortisone (Sigma), estradiol (E2) (Sigma), 300 pM progesterone (P4) (Sigma), and 5 ng/mL epidermal growth factor (EGF) (Sigma). The latter medium is referred to as FBS-free medium. FBS-free medium supplements’ composition was planned according to the most common supplements used for bovine mammary gland epithelial cells in other studies^[41-43]^, with B27 supplementation partially covering a variety of components of FBS. When plates were coated, rat tail collagen I (Invitrogen, USA) at the final concentration of 6 or 10 μg/cm^2^, or laminin (Sigma) at 1 or 2 μg/cm^2^ was used. For the growth curve, 6 × 10^5^ cells were seeded on six-well multiwell plates and counted every two days from Day 4 to Day 14. To evaluate the growth, 6 × 10^5^ cells were seeded on six-well multiwell plates and counted every two days from Day 4 to Day 14 using Trypan blue (Sigma) and a Neubauer chamber.

### MAC-T cell line

MAC-T cells were kindly provided by Olga Wellnitz from the Vetsuisse Faculty of the University of Bern (Switzerland). Cells were cultured in FBS 10% or FBS-free medium and were passaged every 3-4 days. After two weeks of adaptation in the FBS-free medium, they were lysed in TRIzol (Thermo Fisher) to check the expression of cell type markers (keratin 18, keratin 14, and vimentin). For growth rate evaluation, 1 × 10^5^ cells were plated and counted at 80% confluence for two consecutive passages (referred to P1 and P2). The growth rate was calculated as ln(cells t_0_/cells t_1_)/t_1_-t_0_.

### RNA isolation and retrotranscription from cells

Once cells reached 80%-90% confluency, they were washed in PBS and lysed in TRIzol (Thermo Fisher), followed by phenol-chloroform RNA isolation. DNA was removed by DNAse I treatment (Sigma). For each sample, 500 ng of total RNA were reverse transcribed using the GoScript Reverse transcription system kit (Promega, USA), following the manufacturer’s instructions and cDNA samples were then stored at -20 °C until further use. We followed MIQE guidelines for RNA isolation and retrotranscription^[44]^.

### Gene expression analysis

Table 1^[45]^ shows the primer pairs; actin, GAPDH, and histone H3 were used as reference genes, as reported in the literature^[37,46]^. For the RT-qPCR, the Kappa Mix (Sigma) was used and the primer concentration was 10 μM. The amplification was performed using 500 ng of cDNA and the following amplification program: 95 °C for 3 min (×1), 95 °C for 4 s, 60 °C for 20 s, and 95 °C for 10 min (×40). Relative gene expression analysis was performed using the 2^-ΔΔCt^ method^[47]^. We followed MIQE guidelines for gene expression analysis^[44]^ and we used as control groups pbMECs grown in FBS-containing medium without preplating, freshly isolated pbMEC on Day 0, and MAC-T grown in FBS-containing medium, respectively. Actin, Histone H3, and GAPDH were used as reference genes, as reported in other publications^[46,48]^.

**Table 1:** List of the designed forward and reverse primer pairs, KRT14 and PRLR are from the work of Finot *et al.*^[45]^ (2018)

**Gene**	**Forward 5` → 3`**	**Reverse 5` → 3`**
Actin	GTCTTCCCGTCCATCGTG	TCTTGCTCTGAGCCTCATCC
Histone H3	ACTGGCTACAAAAGCCGCTC	ACTTGCCTCCTGCAAAGCAC
GAPDH	GGTCACCAGGGCTGCTTTTA	CCAGCATCACCCCACTTGAT
Keratin 18 (KRT18)	ATTTCAGTCTTGGCGACGCT	GCCTCAGTGCCTCAGAACTT
Vimentin (VIM)	CGCTCAAAGGGACTAACGAG	ACGAGCCATCTCTTCCTTCA
Keratin 14 (KRT14)	CAGCCCCTACTTCAAGACCA	AGGTTCAGCTCCGTCTCGTA
Prolactin hormone receptor (PRLR)	CTTGAAAGGAAGCCAAACAGGC	TGGAGAGAATCAACACCGCC
Progesterone receptor (PR)	GGGACTCTCAGTTCACTTTCAA	TTGTCTGAGTACACGGTGGG
Alpha casein CSN1S1	GGAAGCTGAAAGCATTTCGT	GGGCACATCTTCCTTTTGAA
Kappa casein (CSN3)	TGCAATGATGAAGAGTTTTTTCCTAG	GATTGGGATATATTTGGCTATTTTGT

### Extracellular vesicles isolation from conditioned medium

#### Experimental design

**Figure 1:**
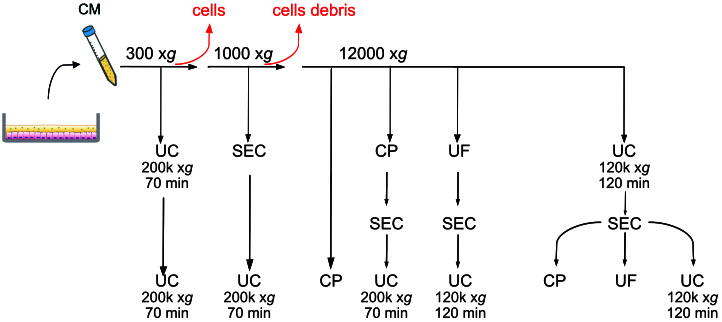
Schematic representation of the eight protocols tested. UC: Ultracentrifugation; UF: ultrafiltration with Amicon tubes; CP: chemical precipitation with miRCURY (Qiagen); SEC: size exclusion chromatography with qEV columns (IZON).

We combined different methods to isolate EVs, testing in total eight protocols, which are schematized in Figure 1. We started from 10 mL of conditioned medium (CM) for all routes except for size exclusion chromatography-ultracentrifugation (SEC-UC) where we started from 0.5 mL.

(1) UC ×2: after the centrifugation at 300 x*g* the supernatant underwent UC at 200,000 x*g* for 70 min. The pellet was resuspended in PBS and spun again at 200,000 x*g*. (2) SEC-UC: after the differential centrifugation at 300 x*g* and 1000 x*g*, 0.5 mL of supernatant were loaded onto a qEV column. Fractions 6-10 were centrifuged at 200,000 x*g* for 70 min. (3) Chemical precipitation (CP): after the differential centrifugation at 300 x*g*, 1000 x*g*, and 12,000 x*g*, the supernatant was precipitated using the miRCURY kit. (4) CP-SEC-UC: after the differential centrifugation at 300 x*g*,1000 x*g*, and 12,000 x*g*, the supernatant was precipitated with the miRCURY kit, the precipitate was loaded on a qEV column, and Fractions 6-10 were spun at 200,000 x*g* for 70 min. (5) UF-SEC-UC: after differential centrifugation, the supernatant was loaded onto an Amicon Tube for ultrafiltration. The concentrate was loaded on a qEV column, and Fractions 6-10 were spun at 120,000 x*g* for 120 min. (6) UC-SEC-CP: after differential centrifugation, the supernatant was centrifuged at 120,000 x*g* for 120 min, the pellet resuspended in 0.5 mL of PBS, and loaded onto qEV column. Fractions 6-10 were precipitated with a miRCURY kit. (7) UC-SEC-UF: after differential centrifugation, the supernatant was centrifuged at 120,000 x*g* for 120 min, and the pellet resuspended in 0.5 mL of PBS and loaded onto qEV column. Fractions 6-10 were loaded on an Amicon Tube for ultrafiltration and the concentrate collected as final EVs suspension. (8) UC-SEC-UC: after differential centrifugation, the supernatant was centrifuged at 120,000 x*g* for 120 min, and the pellet resuspended in 0.5 mL of PBS and loaded onto qEV column. Fractions 6-10 were again spun at 120,000 x*g* for 120 min.

### Differential centrifugation of cells derived conditioned medium

The CM from cells cultured in FBS-free conditions was collected at 80%-90% confluency. The CM was differentially centrifuged at 300 x*g*, 1000 x*g*, and 12,000 x*g* for 10 min at 4 °C to remove dead cells, cells debris, and apoptotic bodies, respectively. Immediately, the CM was frozen at -80 °C until the EV isolation.

### Ultracentrifugation for cultured cells derived EVs isolation

The CM was thawed on ice and transferred into 13.5 mL Ultra-Clear ultracentrifuge tubes (Beckman Coulter, USA). Then, they were spun at 200,000 x*g* for 70 min or 120,000 x*g* for 120 min at 4 °C in a Beckman Coulter Ultracentrifuge Optima XE-90, using a Type 50.2 Ti rotor. The pellets were resuspended in 0.22 μm filtered PBS for further ultracentrifugation, or size exclusion chromatography, and stored at -80 °C.

### Ultrafiltration for cultured cells derived EVs isolation

To perform ultrafiltration (UF), 10 mL of CM or qEV Fractions 6-10 diluted in PBS were loaded on 15 mL Amicon Tubes with a cut-off of 100 kDa (Merck, Germany) and spun for 1 h RT at 5000 x*g* on a FA-45-6-30 fixed angle rotor. The concentrate was transferred into a new tube and either frozen or loaded onto a qEV column (IZON Science Ltd, New Zealand).

### Precipitation with miRCURY (Qiagen) for cultured cells derived EVs isolation

To chemically precipitate the EVs, we used the miRCURY kit (Qiagen, Germany) for cell culture medium, following the manufacturer’s instructions. Briefly, the CM after differential centrifugation or from size exclusion chromatography was spun at 3000 x*g* at 4 °C for 5 min to remove debris and cryo-precipitated particles. The supernatant was transferred into a new tube where 0.4 of sample volume of buffer B was added. After vortexing, the tubes were left on ice for 1 h and then spun at 20 °C at 3200 x*g* for 30 min. The precipitate was resuspended in 100 μL of resuspension buffer and either snap-frozen or loaded onto qEV columns.

### Size exclusion chromatography for cultured cells derived EVs isolation

We performed size exclusion chromatography with qEV 70 nm classic columns (IZON). The columns were first equilibrated at room temperature (RT) with 10 mL of 0.22 μm filtered PBS (Gibco), and then 500 μL of CM or EV resuspension from UC or UF was loaded on the column and eluted in filtered PBS. The flow-through was collected in 500 μL fractions and in each fraction the protein concentration was measured by the A280 at the Nanodrop. Fractions 6-10 were pooled together for further concentration.

### Milk EVs isolation

The milk EVs were isolated according to the protocol described by Somiya *et al.*^[49] ^with some modifications. Briefly, whole milk was centrifuged at 300 x*g*, 3000 x*g*, and 12,000 x*g* to remove, respectively, cells, fat, and apoptotic bodies. Then, 25 mL of skim milk were heated at 37 °C for 10 min, and 1% of acetic acid was added to precipitate the casein micelles and other proteins. The samples were centrifuged at 10,000 x*g* for 10 min at 4 °C, the supernatant was filtered through 0.22 μm and samples were ultracentrifuged at 210,000 x*g* for 70 min at 4 °C (Optimax 90XE, Beckman Coulter). The pellet was washed with PBS and spun again at 210,000 x*g* for 70 min at 4 °C. Finally, the pellet was resuspended with 500 μL of PBS and centrifuged at 10,000 x*g* for 5 min at 4 °C. The supernatant aliquoted and stored at -80 °C was collected.

### Tunable resistive pulse sensing measurements

All measurements were conducted using a qNano Gold (IZON) and NP100 or NP150 polyurethane nanopores (IZON), which detect particles with diameters ranging 50-330 and 70-420 nm, respectively. Filtered PBS (Gibco) was used as an electrolyte buffer and CPC100 (IZON) as calibration particles. Analyses were performed with Izon Control Suite v.3.3.

### Transmission electron microscopy

The EVs visualization was performed at the Scientific Center for Optical and Electron Microscopy service of ETH Zurich. Briefly, 3 μL of the vortexed dispersion were placed on glow discharged carbon-coated grids (Quantifoil, D) for 1 min. Negative contrast staining was done in 2% sodium phosphotungstate pH 7.2 for 1 s, followed by a second step for 15 s. Excess moisture was drained with filter paper and the imaging of the air-dried grids was done in a transmission electron microscopy (TEM) Morgagni 268 (Thermo Fisher) operated at 100 kV. For each experimental group, two replicates were analyzed.

### Protein isolation and Western blot analysis

The pellet of freshly isolated EVs from 45 mL of medium was immediately lysed in RIPA buffer plus protease inhibitors and then stored at -80 °C. To each sample, 4× Laemmli buffer (Biorad, USA) was added and heated for 10 min at 95 °C. Between 1-3 μg of proteins (for bMEC EVs, milk EVs, and cell lysates) were run on 12% polyacrylamide gel and then total protein was evaluated at the Chemidoc running the stain-free program. Proteins were transferred using a TransTurbo transfer pack (Biorad) with a TurboBlot (Biorad). Membranes were blocked for 1 h in skim milk 5% TBS-Tween buffer (TBST, Bio-Rad, 0.05% Tween 20), and incubated overnight with the primary antibodies diluted in blocking buffer: anti-TSG101 (1:250, PA531260, Thermo scientific), anti-calnexin (1:2000, ab75801, Abcam, UK), and anti-CD9 (1:250, MM2/57, Biorad). After three washes in TBST, membranes were incubated for 1 h at RT with secondary antibodies (Santa Cruz Biotechnology, USA) and StrepTactin-AP Conjugate (Biorad) at the concentration of 1:10,000 and eventually incubated with Clarity Western ECL Substrate (Biorad) for chemiluminescent signal development.

### RNA isolation from EVs

Total RNA including microRNA (miRNA) was isolated with the miRNeasy MicroKit (Qiagen, Germany). The length from RNA fragments was evaluated using the Agilent Pico Kit and the Agilent 2100 BioAnalyzer (Agilent Technologies).

### EVs RNA retrotranscription and RT-qPCR

For each sample, 1 ng of RNA was reverse transcribed and pre-amplified using TaqMan™ Advanced miRNA assays (Life Technologies), following the manufacturer’s instructions. Then, we performed an RT-qPCR using kit TaqMan™ Advanced miRNA assays targeting let-7a-5p (478575_mir assay), miR-200c-3p (mmu482938_mir assay) and miR-223-3p (477983_mir assay). The amplification program was: 95 °C for 30 s (×1), 95 °C for 5 s, and 60 °C for 30 s (×40).

### Statistical analysis

To evaluate the gene expression of mammary epithelial markers, we performed the non-parametric Friedman test and Dunn’s multiple comparisons tests using GraphPad Prism version 8.2. The differences were considered significant when *P* < 0.05. Unless stated, all values are given as mean ± standard deviation (SD).

## RESULTS

### Evaluation of cells growth in FBS-free medium

**Figure 2:**
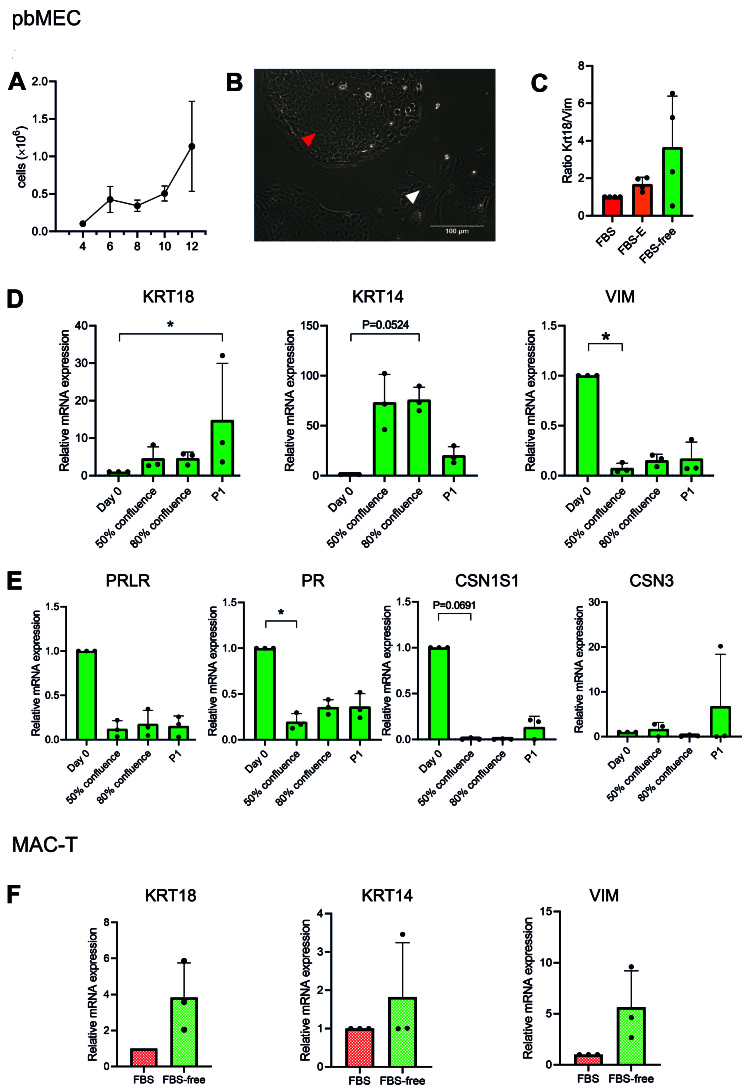
The pbMEC and MAC-T properties in FBS-free medium. (A) Cell count of pbMEC from Day 4 to Day 12 after the isolation from fresh tissue, in six-well multiwell culture dishes. (B) The picture of cultured pbMEC shows the heterogeneous population of epithelial cells (red arrow) and fibroblast-like cells (white arrow). Scale bar: 100 μm. (C) Enrichment of epithelial cells over fibroblasts as means of mRNA expression of keratin 18 (KRT18)/vimentin (VIM) in FBS-containing medium with or without preplating and FBS-free medium. (D, E) mRNA expression in pbMECs of cell-type (D) and differentiation (E) markers on the isolation day (Day 0), at 50% and 80% confluency, and at 80% confluency at the first sub-passage (P1): keratin 18 (KRT18), keratin 14 (KRT14) for epithelial identity, vimentin (VIM) as fibroblastic marker, prolactin hormone receptor (PRLR), progesterone receptor (PR), casein alpha (CSN1S1) and casein kappa (CSN3) coding genes as differentiation markers. (F) Keratin 14 and 18 and vimentin mRNA expression for MAC-T cells cultured in FBS 10% or FBS-free medium. The mRNA expression data are depicted as 2^-ΔΔCt^^[47]^, values are mean ± SD of four independent replicates, and Kruskal-Wallis and Dunn’s multiple comparisons tests (C-E) or Wilcoxon test (F) were performed. The differences were considered significant when *P* < 0.05, (marked with “*” on the graph). pbMEC: Primary bovine mammary epithelial cells; FBS: fetal bovine serum.

From the isolation (Day 0) to 80% confluency, pbMECs took on average 18.5 ± 4.07 days when grown in FBS-free medium. Cells grew from ~1 × 10^5^ on Day 4 to ~1.1 × 10^6^ (Day 12) [Figure 2A]. Coating the plates with collagen I or laminin improved neither cell attachment nor growth during the first days [Supplementary Figure 1A]; therefore, we kept plating on uncoated wells. The pbMECs were cultured over three passages in FBS-free medium. MAC-T cells grew in FBS-free medium as well, and the growth rate did not change compared to FBS-containing medium (*P* > 0.05, Table 2).

**Table 2:** Growth rates of MAC-T grown in FBS-containing or FBS-free medium in two consecutive passages

**MAC-T cells**	**Passage 1 (h^-1^)**	**Passage 2 (h^-1^)**
FBS	0.011 ± 0.006	0.023 ± 0.003
FBS-free	0.022 ± 0.015	0.012 ± 0.005

The formula growth rate = ln(cells t0/cells t1)/t1-t0 was used. The time frames from t0 to t1 were between 72 and 216 h. Data are expressed as mean ± SD of three independent experiments. Mann-Whitney test was performed. FBS: Fetal bovine serum.

### Evaluation of mammary gland gene markers expression in FBS-free medium

The primary cultures were initially not pure and were composed of a mixed population of fibroblasts (Figure 2B, white arrow) and epithelial cells (Figure 2B, red arrow). We evaluated the epithelial cells’ enrichment as indicated by the gene expression ratio between keratin 18 (an epithelial marker) and vimentin (a fibroblastic marker) in pbMECs cultured in: (1) FBS 10%; (2) FBS 10% + preplating; and (3) FBS-free medium. The enrichment of epithelial cells increased 1.7-fold by preplating and 3.4-fold in FBS-free medium [Figure 2C], compared to pbMECs in FBS without preplating. Even if not statistically significant (*P* = 0.1377, FBS-free medium* vs.* FBS 10%), we observed that some cultures reacted to the FBS-free medium by enriching the population in epithelial cells, without the need of preplating. Coating the plate with neither collagen I nor laminin significantly affected the enrichment (*P* > 0.05, Supplementary Figure 1B).

Subsequently, we evaluated the gene expression of cell type and differentiation markers at Day 0 (isolation day), 50% and 80% confluence after day 0, and at 80% confluence after the first sub-passage (P1). The mRNA abundance of the epithelial markers keratin 14 (KRT14) and 18 (KRT18) increased at 80% confluency after the isolation day and at P1, respectively (*P* < 0.05, Figure 2D). On the other hand, the expression of the fibroblastic marker vimentin (VIM) decreased already at 50% confluence after day 0 (*P* < 0.05, Figure 2D). The expression of the functional marker progesterone receptor (PR) significantly decreased already at 50% confluence (*P* < 0.05, Figure 2E), while casein alpha (CSN1S1) and prolactin hormone receptor (PRLR) tended to decrease over time (*P* > 0.05, Figure 2E). Casein kappa (CSN3) did not significantly change, but the trend was rather to increase in one replicate [Figure 2E].

MAC-T cells cultured in FBS-free medium presented as a homogeneous population and did not morphologically differ from the FBS-containing environment [Supplementary Figure 1C]. Gene expression of KRT14, KRT18 and VIM did not change (*P* > 0.05, Figure 2F), indicating that the FBS-free medium did not alter the cell-type composition.

### Evaluation of EVs isolation methods from pbMECs and MAC-T conditioning medium

**Figure 3:**
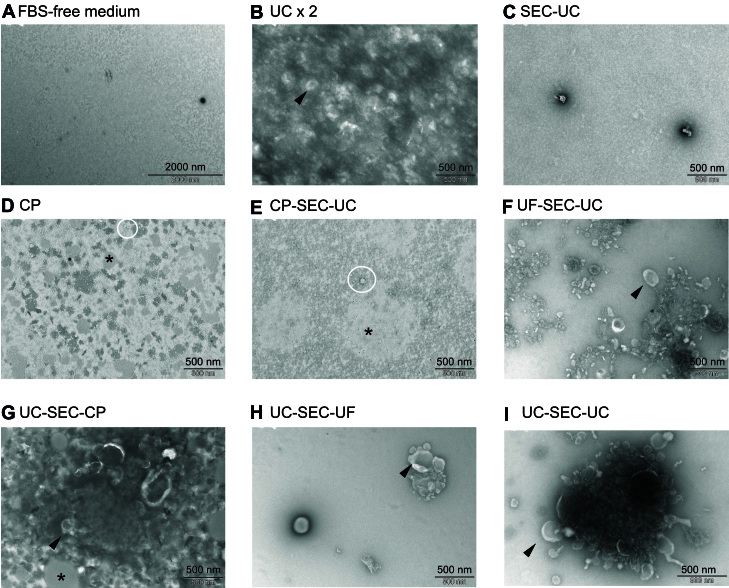
Electron microscopy pictures from FBS-free medium (A) and EVs isolated with UC ×2 (B), SEC-UC (C), CP (D), CP-SEC-UC (E), UF-SEC-UC (F), UC-SEC-CP (G), UC-SEC-UF (H), and UC-SEC-UC (I). Black arrow: cup-shaped particles (vesicles); white circles: non-vesicles particles; black asterisks: undefined aggregates. FBS: Fetal bovine serum; EVs: extracellular vesicles; UC: ultracentrifugation; SEC: size exclusion chromatography; CP: chemical precipitation.

As shown in Figure 3A, the conditioning FBS-free medium alone did not contain any vesicles, ruling out any contamination of EVs from the medium used. According to TEM, we obtained particles from all routes except from SEC-UC [Figure 3C].

Single or aggregates of cup-shaped particles exhibiting the typical EV morphology in TEM^[50-53] ^were observed following UC ×2, UF-SEC-UC, UC-SEC-UC, UC-SEC-UF, and UC-SEC-UC (Figure 3B and F-I black arrows). The observed structures were heterogeneous in size. We also observed light grey aggregates in CP, CP-SEC-UC, and UC-SEC-CP (Figure 3D, E and G; black asterisk), likely due to lipid aggregates^[54,55]^, while round non-vesicles^[56]^ were observed from CP, CP-SEC-UC, and UF-SEC-UC (Figure 3D and E; white circles). Size and concentration of the isolated particles are indicated in Supplementary Table 1.

**Figure 4:**
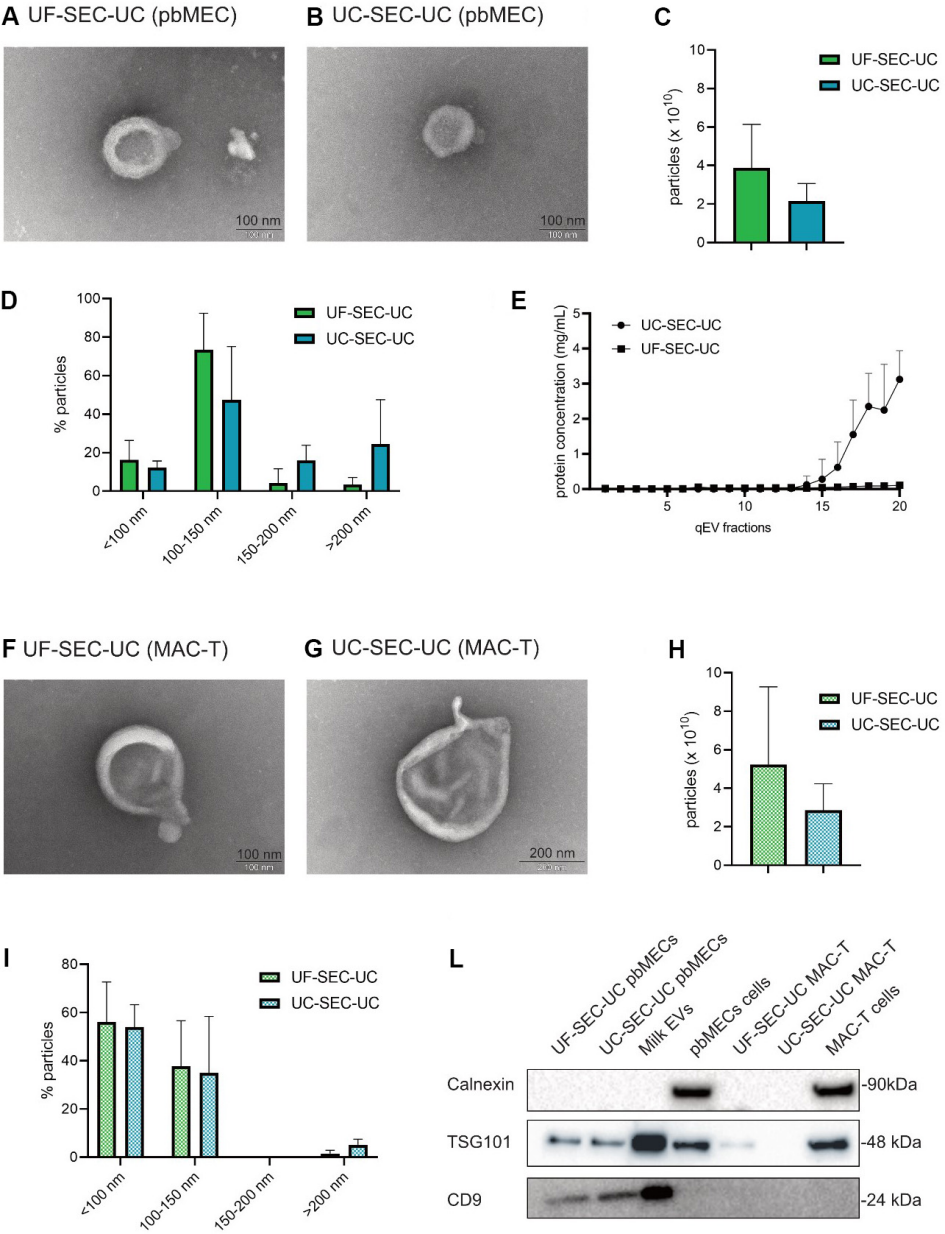
Characterization of the EVs from UF-SEC-UC and UC-SEC-UC in pbMEC and MAC-T (A, B) electron microscopy picture of a vesicle from UF-SEC-UC (A) and UC-SEC-UC (B) on pbMEC conditioned medium. (C, D) Total number of particles per 10 mL of conditioned medium (C) and size ranges (D) from EVs from UF-SEC-UC and UC-SEC-UC in pbMEC conditioned medium. The TRPS was performed with a NP100 nanopore. (E) Protein concentration of the single fractions from SEC from pbMECs conditioning medium. (F, G) Electron microscopy pictures of vesicles from UF-SEC-UC (F) and UC-SEC-UC (G) on MAC-T conditioned medium. (H, I) Total number of particles per 10 mL of conditioned medium (H) and size ranges (I) from EVs from UF-SEC-UC and UC-SEC-UC in pbMEC conditioned medium. The TRPS was performed with a NP100 nanopore. (L) Western blot of pbMECs and MAC-T EVs pellet. Whole cells lysates were used as a positive control for calnexin, while EVs from milk were used as a positive control for EV markers. Full-length blots are presented in Supplementary Figure 2. In (C-E, H, I), values are mean ± SD of three replicates. Wilcoxon test (C, H) was performed. The differences were considered significant when *P* < 0.05. FBS: Fetal bovine serum; EVs: extracellular vesicles; UC: ultracentrifugation; SEC: size exclusion chromatography; pbMEC: primary bovine mammary epithelial cells; UF: ultrafiltration.

Since UF-SEC-UC and UC-SEC-UC had a clean background free of undefined aggregates [Figure 3F and I], and a high number of particle, we selected these two routes to perform tunable resistive pulse sensing (TRPS) analysis and RNA extraction. Figure 4A and B shows at higher magnification the isolated particles. We measured by TRPS the number of particles and their size. We obtained similar numbers of particles [Figure 4C] as well as size distributions [Figure 4D] across replicates (*P* > 0.05, Figure 4C). The last SEC fractions after UF had lower protein concentration than after UC, showing that UF as a first step better cleaned the samples from contaminating proteins [Figure 4E].

When isolating EVs from MAC-T with UF-SEC-UC and UC-SEC-UC, we obtained in both routes cup-shaped particles as shown with TEM [Figure 4F and G]. The TRPS measurement showed that the number of isolated particles [Figure 4H] and size distributions [Figure 4D and I] were similar (*P* > 0.05) between the two protocols.

In pbMEC conditioning medium, the isolated particles were positive for both the EVs markers, TSG101 and CD9, while in MAC-T conditioning medium it was only positive for TSG101 from UF-SEC-UC. All lysates were negative for calnexin, ruling out any intracellular contamination^[57] ^[Figure 4L].

**Figure 5:**
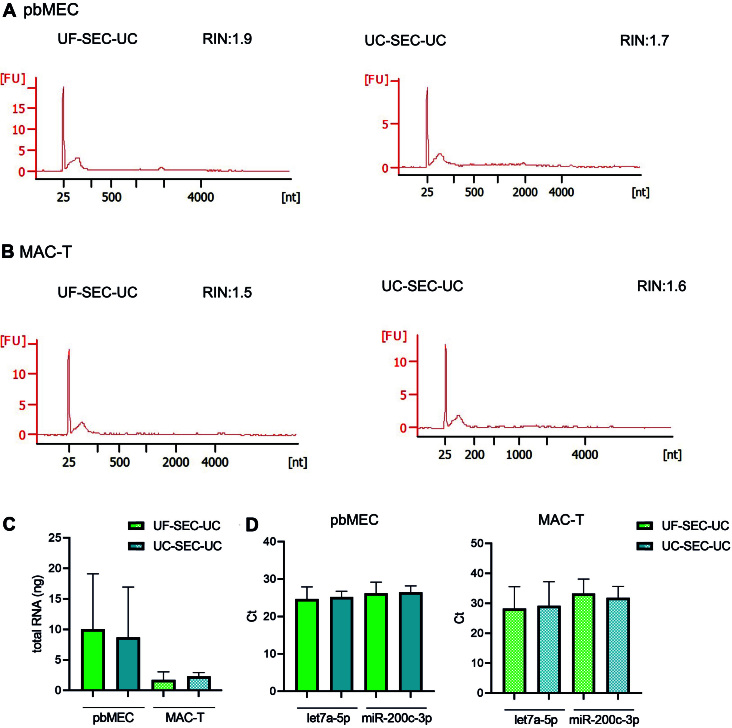
Isolation and characterization of RNA from pbMEC and MAC-T EVs. (A, B) Representative Agilent 2100 Bioanalyzer data from pbMEC (A) or MAC-T (B) RNA from EVs isolated by UF-SEC-UC (right) and UC-SEC-UC (left). (C) Total amount of RNA from pbMECs and MAC-T EVs isolated with UF-SEC-UC or UC-SEC-UC. (D) qPCR of let7a-5p and miR-200c-3p from EVs RNA isolated with either UF-SEC-UC or UC-SEC-UC from pbMEC (left) or MAC-T (right). (C, D) Bars show mean and SD of three independent experiments. EVs: Extracellular vesicles; UC: ultracentrifugation; SEC: size exclusion chromatography; pbMEC: primary bovine mammary epithelial cells; UF: ultrafiltration.

We isolated detectable amounts of RNA from both pbMEC and MAC-T EVs. The length of the isolated RNA molecules is displayed in Figure 5A and B and in Supplementary Figure 3. Specifically, we obtained on average 10.6 ± 9.1 ng from UF-SEC-UC and 8.6 ± 8.2 ng from UC-SEC-UC in pbMECs, while in MAC-T 1.7 ± 1.3 and 2.4 ± 0.6 ng, respectively [Figure 5C]. Finally, the isolated EVs from both pbMECs and MAC-T contained the miRNA let7a-5p and miR-200c-3p. The protocol for EV isolation did not affect the amount of detected miRNA [Figure 5D]. On the other hand, we could detect miR-223c-5p only at Cts higher than 35 (not shown in the Figure).

## DISCUSSION

In the current study, we established for the first time the culture of primary bovine mammary epithelial cells (pbMECs) and MAC-T cells in FBS-free medium to study EVs from the bovine mammary gland* in vitro*. An important point is that the culture medium is chemically defined, therefore it does not change during the culture and always keeps the same formula, avoiding any possible source of variability given by FBS removal^[28,29]^ or even different batches of FBS.

Our customized FBS-free medium sustained pbMEC growth until confluency and beyond Passage 3, as well as the growth of the MAC-T cell line. The growth rate of MAC-T was lower than in FBS-containing medium. This is in line with previous results, where, however, the culture medium had a different formula^[58]^. In the primary culture, FBS-free medium promoted the expression of epithelial markers keratin 14 and 18 and the downregulation of the fibroblastic marker vimentin, thereby enriching the population in epithelial cells. In addition, it did not affect the epithelial identity of MAC-T, as keratin 14 and 18 expression levels did not change. Thus, pbMECs and MAC-T cells cultured on FBS-free medium were still relatively close to the primary isolated cells at Day 0 and FBS-containing medium, respectively.

We also observed a downregulation of the differentiation markers PR, PRLR, CSN1S1, and CSN3 already starting at 50% confluency. This trend to de-differentiate might be due to the two-dimensions (2D) culture on plastic dishes, which by itself promotes de-differentiation^[42,59]^. It has been shown that the growth of pbMECs on three-dimensional (3D) systems supports differentiation^[37,42,60]^. Thus, further studies should focus on 3D FBS-free culture systems.

The most commonly used techniques to isolate EVs from primary MECs cell cultures or breast cancer lines are UC ×2^[6,18,23,61,62]^ and UF with a cut-off of 100 kDa^[17]^, respectively. By combining these methods, together with size exclusion chromatography and chemical precipitation, we managed to isolate EVs from relatively low amounts (10 mL) of cellular conditioning medium, while in the literature, when stated, the reported starting material is often between 20 and 250 mL^[30-32]^. The TEM and TRPS measurements excluded any particle contamination in the FBS-free medium, thereby the EVs we observed and analyzed were secreted by the cultured cells. From the eight protocols that we tested, only in one protocol (SEC-UC) we did not isolate any particle after TEM analysis, likely due to the initial low amount of starting material (500 μL). In UC ×2, TEM imaging showed that the matrix was not clean, possibly due to the low number of cleaning steps before the UC. The introduction of more differential centrifugation steps and a SEC step helped to clean the sample likely from proteins in solution and any membranes or content deriving from cell debris and apoptotic bodies, as the CM underwent only centrifugation at 300 x*g*. The miRCURY kit used for CP and CP-SEC-UC instead helped to precipitate vesicles but concomitantly generated many undefined aggregates, as the precipitation itself does not distinguish between the types of macromolecules in solution^[33,55]^. We obtained a clean sample from UC-SEC-UF, but the particles observed after TEM were few in the whole grid, likely due to the high final volume of the sample (150 μL), collected from the ultrafiltration tube. Both UF-SEC-UC and UC-SEC-UC revealed a better compromise regarding the yield and purity of vesicles. In both pbMEC and MAC-T, the TRPS measurements confirmed the size ranges observed with TEM and were similar between routes, and both gave a consistent number of EVs, on the order of 10^10^ particles from 10 mL of medium, which tended to be higher from UF-SEC-UC. Due to the nanopore size used in the TRPS analysis, we could not detect particles smaller than 50 nm, likely excluding many EVs observed by TEM and therefore underestimating the real concentration of the sample.

Western blot analysis confirmed that the isolated vesicles were actual EV, bearing both CD9 and TSG101, in line with the results obtained by Zhang *et al.*^[17] ^and from studies on milk EVs^[19]^. We speculate that the faint TSG101 band for MAC-T EVs and absence of CD9 might be due to the low input volume of conditioned medium, as the presence of these two markers in MAC-T exosomes was reported^[63]^. Importantly, the use of a SEC step to separate secreted proteins from EVs would allow analyzing the cell response, distinguishing the contribution of EVs and secreted proteins. For such purpose, UC-SEC-UC would be more suitable, as the protein-rich fractions are more concentrated, without the initial UF step. On the other hand, if the experimental setup requires the study of EVs only, UF-SEC-UC would be more recommended, as the sample is initially depleted from proteins smaller than 100k kDa.

We were able to extract RNA from both UF-SEC-UC and UC-SEC-UC in pbMEC as well as MAC-T EVs, and the amount of isolated RNA did not differ significantly among conditions. All samples showed the typical profile of exosomal RNA , ruling out any contamination from apoptotic bodies^[64]^. We also excluded a possible contamination from milk fat globule RNA^[65]^, as the cultured cells expressed very low levels of caseins, indicating low or no milk production. The two miRNAs let-7a-5p and miR-200c-3p were reported as some of the most abundant miRNAs in milk exosomes^[66]^ as well as in cultured pbMEC exosomes^[67]^. In addition, the no detection of miR-223-3p is also in line with the literature, as this miRNA is not reported among the most abundant^[66]^ and it is only upregulated upon infection^[4,5]^.

In conclusion, we demonstrated that the FBS-free medium culture system is a valid tool to study MEC EVs from both primary cells and the MAC-T cell line. We evaluated and compared different EVs isolation protocols from a relatively low amount of starting cell culture medium (10 mL), and we propose UF-SEC-UC as the preferred method as it yields the highest number of EVs and pure EVs for further downstream analysis in both pbMEC and MAC-T. Our results provide an important reference for further studies that aim at analyzing MEC EVs in many contexts such as lactation, infection, response to stressors and metabolic challenges.

## DECLARATIONS

### Copyright

© The Author(s) 2021.
