# Supplementary material for: Assessing extracellular vesicles from bovine mammary gland epithelial cells cultured in FBS-free medium
Source: Extracell Vesicles Circ Nucl Acids. 2021 Dec 31;2(4):252–67. doi: 10.20517/evcna.2021.18 (PMC11648452; doi:10.20517/evcna.2021.18)
Supplement: Supplementary file 1 [file evcna-2-4-252-SupplementaryMaterials.pdf]

Supplementary Material: Assessing extracellular vesicles from bovine mammary gland epithelial cells cultured in FBS-free medium

Supplementary Figure 1

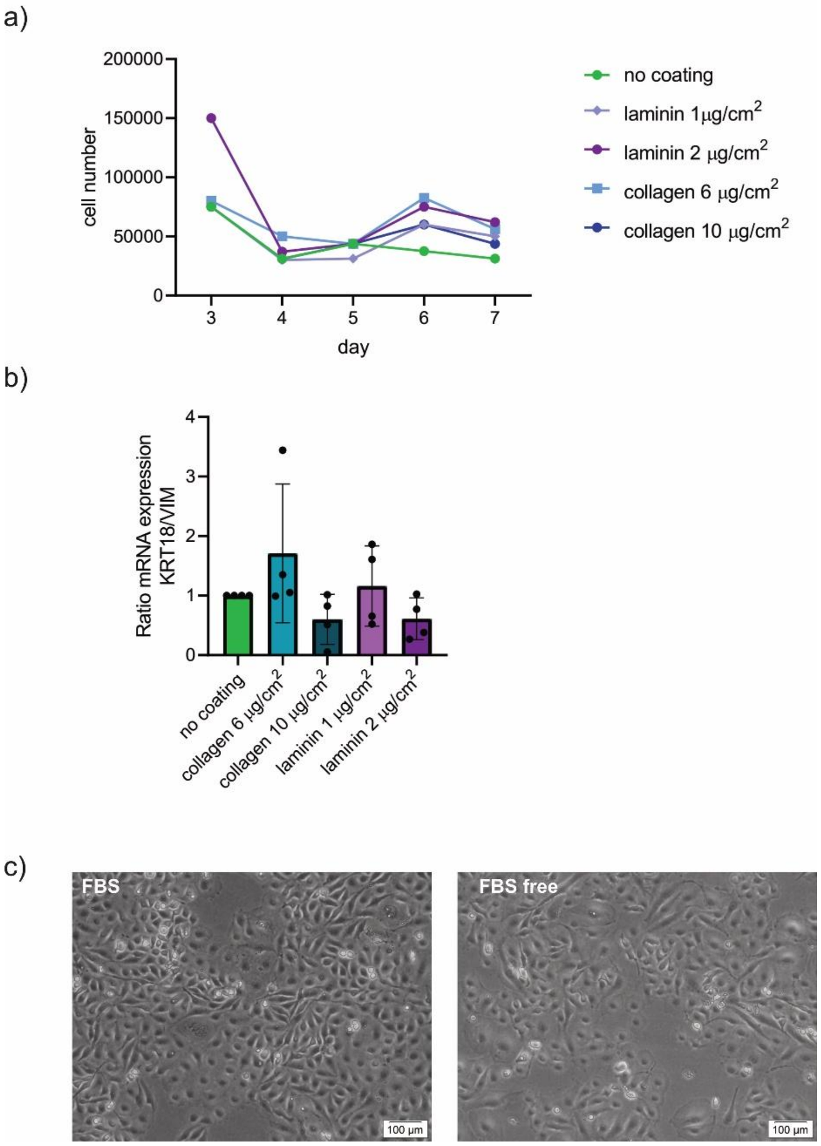

Supplementary Figure 1.. a) Growth curve of pbMECs in FBS-free medium. Cells were plated on 6-well multiwell dishes and counted every day from day 3 to day 7; the culture dishes were uncoated or coated with laminin 1 or 2 µg/cm2 or collagen I 6 or 10 µg/cm2; b) Ratio of the mRNA expression of KRT18/VIM of pbMECs at 80% confluence plated in coated or coated wells as in a); c) MAC-T cells grown in FBS 10% or FBS-free medium.

© The Author(s) 2023. Open Access This article is licensed under a Creative Commons Attribution 4.0 International License (https://creativecommons.org/licenses/by/4.0/), which permits unrestricted use, sharing, adaptation, distribution and reproduction in any medium or format, for any purpose, even commercially, as long as you give appropriate credit to the original author(s) and the source, provide a link to the Creative Commons license, and indicate if changes were made.

Supplementary Figure 2

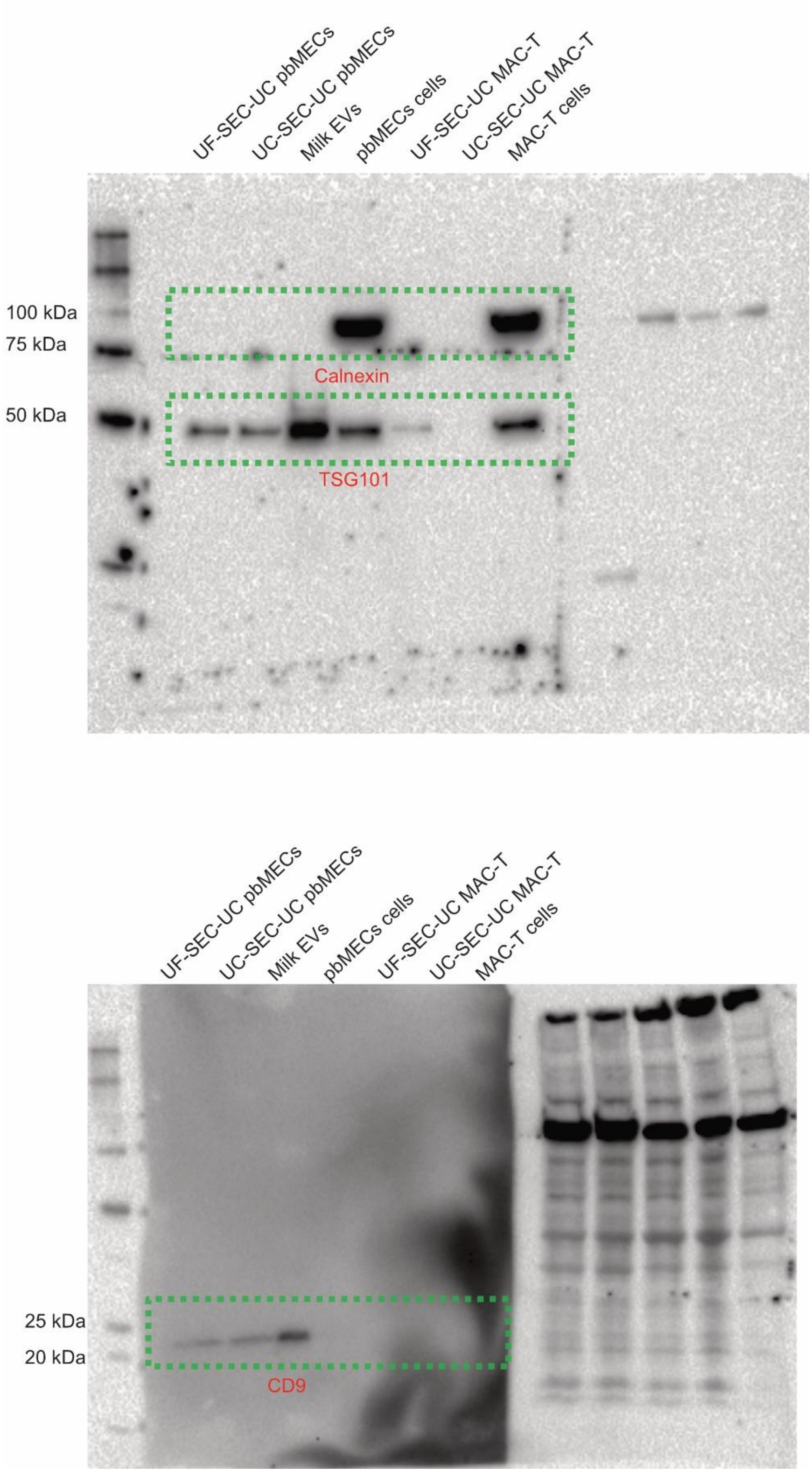

Supplementary Figure 2. Full-length western blots shown in figure 4c. The selected areas are in green.

Supplementary Figure 3

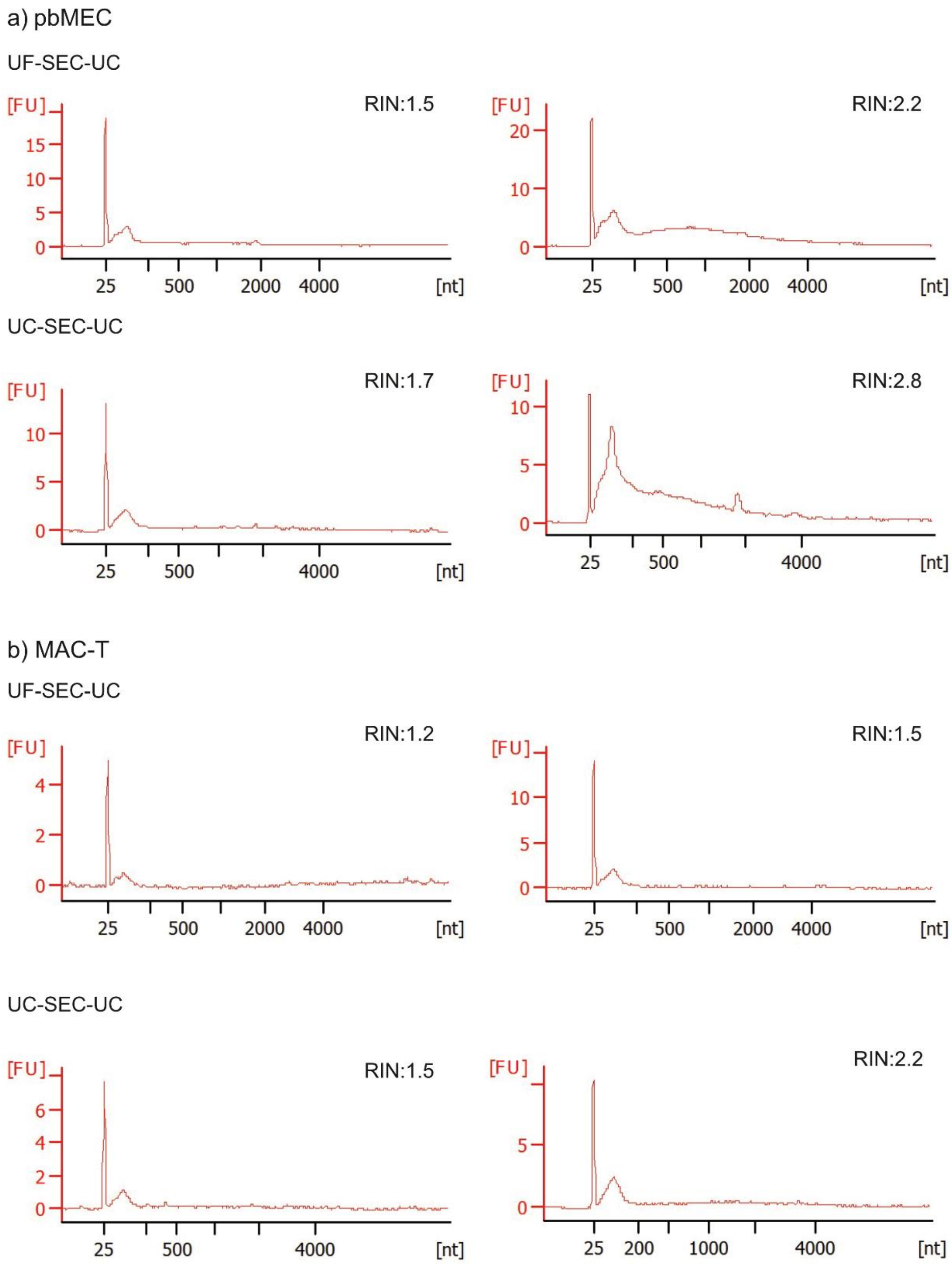

Supplementary figure 3. Agilent 2100 Bioanalyzer data from EVs RNA isolated from pbMEC (a) or MAC-T (b) with either UF-SEC-UC or UC-SEC-UC

Supplementary Table 1

Table 1. Summary of TRPS measurements. The data come from one replicate and were measured with a nanopore NP150.

Route	Pellet resuspension volume [µL]	Raw concentration [particles/mL]	Size range	Mean particle size [nm] ±SD	Particle rate [particles/min]
UCx2	30	4.39E+11	67-512	119 ± 39.7	192.8
SEC-UC	30	-	-	-	-
CP	100	2.95E+09	108-781	195 ± 70.1	119.5
CP-SEC-UC	100	4.99E+08	123-708	210 ± 89.4	20.02
UF-SEC-UC	30	5.30E+10	70-473	139 ± 36.5	277.4
UC-SEC-CP	30	1.47E+10	81-564	151 ±70.0	701.2
UC-SEC-UF	150	-	-	-	-
UC-SEC-UC	30	6.87E+09	77-559	158 ± 60.8	327.0
